# Epidemiology of neuropsychiatric symptoms and AD/ADRD risk and survival in older U.S. adults

**DOI:** 10.1093/geroni/igaf122.1562

**Published:** 2025-12-31

**Authors:** Igor Akushevich, Arseniy Yashkin, Larry Tupler, Meishuo Ouyang, Julia Kravchenko

**Affiliations:** Duke University, Durham, North Carolina, United States; Duke University, Durham, North Carolina, United States; Duke University, Durham, North Carolina, United States; Duke University School of Medicine, Durham, North Carolina, United States; Duke University School of Medicine, Durham, North Carolina, United States

## Abstract

Alzheimer’s disease and related dementias (AD/ADRD) pose a critical public health challenge, further complicated by the high prevalence of neuropsychiatric symptoms (NPS). Using a 5% Medicare sample, we quantified the epidemiology of NPS in relation to AD/ADRD onset and survival. We identified the onset dates for nine NPS—aggression, delirium, wandering, restlessness/agitation, hallucinations, anxiety, demoralization/apathy, sleep disturbances, and psychosis—and estimated their prevalence, the hazard ratios for developing AD and non-AD ADRD, and the hazard ratios for mortality following AD/ADRD diagnoses. We found that prevalence increases with age; maximum values at AD diagnosis were detected for anxiety (38.3%), psychosis (33.1%), and delirium (17.6%). Hazard ratios for AD/ADRD risk were evaluated using the Cox model with predictors measured with a one-year lag to avoid accounting ties between NPS and AD/ADRD onsets. The highest effects were detected for restlessness and agitation (HR = 4.30, CL = 4.21-4.39), psychosis (4.16; 4.12-4.20), and demoralization and apathy (4.15; 3.28-5.26). The effect of any NPS was 2.47 (2.45-2.48). Finally, we calculated the death hazard ratios for cohorts of individuals with diagnoses of AD and ADRD using a left-truncation design and age as the time-scale variable. The highest effect was detected for restlessness and agitation (1.61; 1.59-1.62) and delirium (1.52; 1.51-1.53). The death hazard ratio for any NPS was (1.46; 1.45-1.47). Our robust and biologically interpretable estimates provide new knowledge on underlying pathological processes—including an improved understanding of NPS and their treatment, and additional clues to potential underlying neuropathology—and to facilitate applications at individual-patient and population levels.

